# Rab27A overexpression promotes bladder cancer proliferation and chemoresistance through regulation of NF-κB signaling

**DOI:** 10.18632/oncotarget.20775

**Published:** 2017-09-08

**Authors:** Jia Liu, Xue Gong, Xingwang Zhu, Dongwei Xue, Yili Liu, Ping Wang

**Affiliations:** ^1^ Department of Urology, The Fourth Affiliated Hospital of China Medical University, Shenyang, China

**Keywords:** Rab27A, bladder cancer, FAK, NF-κB

## Abstract

Overexpression of Rab27A has been found in human cancers. However, the clinical significance and biological effects of Rab27A in bladder cancer tissues and cell lines have not been investigated. Here, we checked Rab27A protein in 87 cases of bladder cancer using immunohistochemistry. We found that Rab27A was overexpressed in 39 of 87 (44.8%) cancer cases. Significant association was found between Rab27 and invading depth (p=0.0083). We knocked down Rab27A in 5637 cell line and transfected Rab27A plasmid in BIU-87 cell line. Rab27A depletion inhibited cell growth rate and invasion while its overexpression induced cell growth and invasion. Rab27A also promoted cancer cell growth *in vivo*. Cell viability and Annexin V/PI staining demonstrated that Rab27A maintained cancer cell survival and reduced apoptosis rate when treated with cisplatin. JC-1 staining showed that Rab27A upregulated mitochondrial membrane potential. Western blot demonstrated that Rab27A overexpression upregulated cyclin D1, cyclin E, p-IκB, p-p65, Bcl-2, cIAP1, cIAP2 protein expression. NF-κB inhibitor BAY 11-7082 abolished the effects of Rab27 on cisplatin resistance and Bcl-2 protein. In conclusion, the present study demonstrated that Rab27A overexpression facilitates bladder cancer growth, invasion and chemoresistance in bladder cancer, possibly through regulation of NF-κB signaling pathway.

## INTRODUCTION

Bladder cancer a common urological carcinoma and its incidence is rising in recent years [1]. In most cases, bladder cancers arise from urothelial transitional cells [2]. Despite significant advances in therapies including surgery and chemotherapy, the overall survival rate is not significantly improved. It is very important to search for the molecular mechanism and potential targets regulating bladder cancer aggressiveness and chemoresistance, which could be used for development of new therapy [3-5].

The Rab27 is a family of small GTPases which could regulate exocytosis [6]. Rab27 was recently implicated in human cancer development. Rab27B has been reported to promote invasion and metastasis of breast cancer cells [7]. In melanoma, Rab27A regulated vesicular trafficking is reported to drive malignant tumor growth [8]. Rab27A depletion decreased exosome production, preventing bone marrow education and reducing, tumor growth and metastasis of melanoma cells [9]. Rab27A mediated by NF-κB promotes the stemness of colon cancer cells via up-regulation of cytokine secretion [10]. Rab27a expression was significantly associated with grade progression and worse prognosis in all grades of gliomas [11]. These studies suggested Rab27A as a potential onco-protein in human cancers. However, several reports indicates Rab27A as a tumor suppressor. High Rab27A expression indicates favorable prognosis in colorectal cancer [12]. RAB27A and RAB27B are also downregulated in advanced prostate cancer [13]. Thus its role is controversial. In addition, its clinical significance and biological roles in human bladder cancers remains unexplored yet.

To clarify the above question, we explored expression of Rab27A in bladder cancer by immunohistochemistry. In this study, we also linked Rab27A with NF-κB signaling, which is closed related to cancer cell proliferation and chemoresistance.

## RESULTS

### Expression of Rab27A in bladder cancer samples

**Figure 1:**
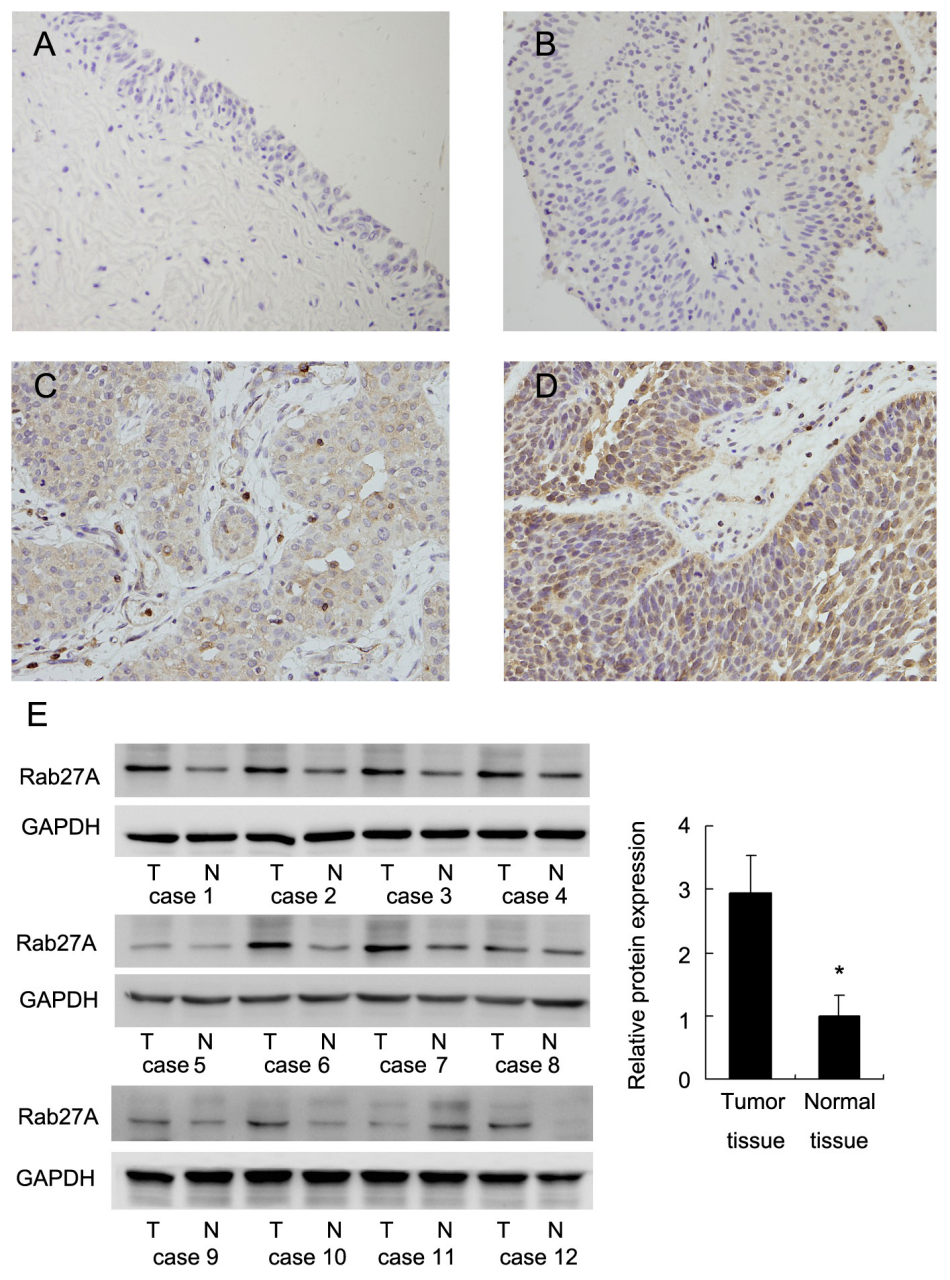
Expression pattern of Rab27A in bladder cancer tissues **(A)** Negative Rab27A staining in the majority of normal bladder epithelial tissues. **(B)** Negative Rab27A staining in a case of non-muscle invasive bladder cancer tissue. **(C)** Weak cytoplasmid Rab27A staining in a case of muscle invasive bladder cancer. **(D)** Strong cytoplasmic/nuclear staining of Rab27A in a case of muscle invasive bladder cancer. (Magnification, 400X). **(E)** Rab27A protein expression was examined in 8 paired tumor (T)/adjacent normal (N) tissues. Western blot revealed that Rab27A overexpression was obvious in 7/12 of these paired tissues. Relative staining intensity was quantified by ImageJ software. The average western blot intensity was higher in cancer tumor tissues compared with that in normal tissues. * p<0.05.

We investigated Rab27A protein in a panel of 87 bladder cancer samples and normal tissues using immunohistochemistry. Rabbit IgG isotype antibody was used for negative control. Tumor samples incubated with Rabbit IgG exhibited negative staining. Rab27A protein was mainly localized in the cytoplasmic compartment of tumor cells. We found negative Rab27A expression in normal urothelial transitional epithelial tissues (Figure 1A). Rab27A staining intensity was divided as negative/weak staining (Figure 1A, 1B), moderate staining (Figure 1C) and strong staining (Figure 1D). Intensity score and percentage score were multiplied to get a final score, which divide RAB27A status as low expression and high expression/overexpression. Rab27A protein high expression/overexpression was observed in 39 out of 87 (44.8%) bladder cancer tissues. As shown in Table 1, statistical significance was found between Rab27A overexpression and local invasive depth/T stage (Ta-T1 vs T2-T4, p=0.0083). The expression rate (63.2%) of Rab27A in muscle invasion bladder cancers (MIBC) was higher than that (34.7%) in non-muscle invasion bladder cancers (NMIBC), suggesting Rab27A is an indicator of muscle invasion bladder cancers. We also examined its protein levels in 12 fresh cancer tissues and paired adjacent normal tissues. Western blot analysis showed significant Rab27A overexpression in 7/12 of paired bladder cancer tissues (Figure 1E).

**Table 1:** Distribution of Rab27A in bladder cancer according to clinicopathological characteristics

Characteristics	Number of patients	Rab27A low expression	Rab27A high expression	*P*
Age				
<60	38	22	16	0.4087
≥60	49	24	25	
Gender				
Male	60	31	29	0.7367
Female	27	15	12	
Tumor status				
Ta-T1	49	32	17	0.0083
T2-T4	38	14	24	
Tumor grade				
G1	37	22	15	0.2898
G2-G3	50	24	26	

### Rab27A promotes proliferation *in vitro* and *in vivo*

**Figure 2:**
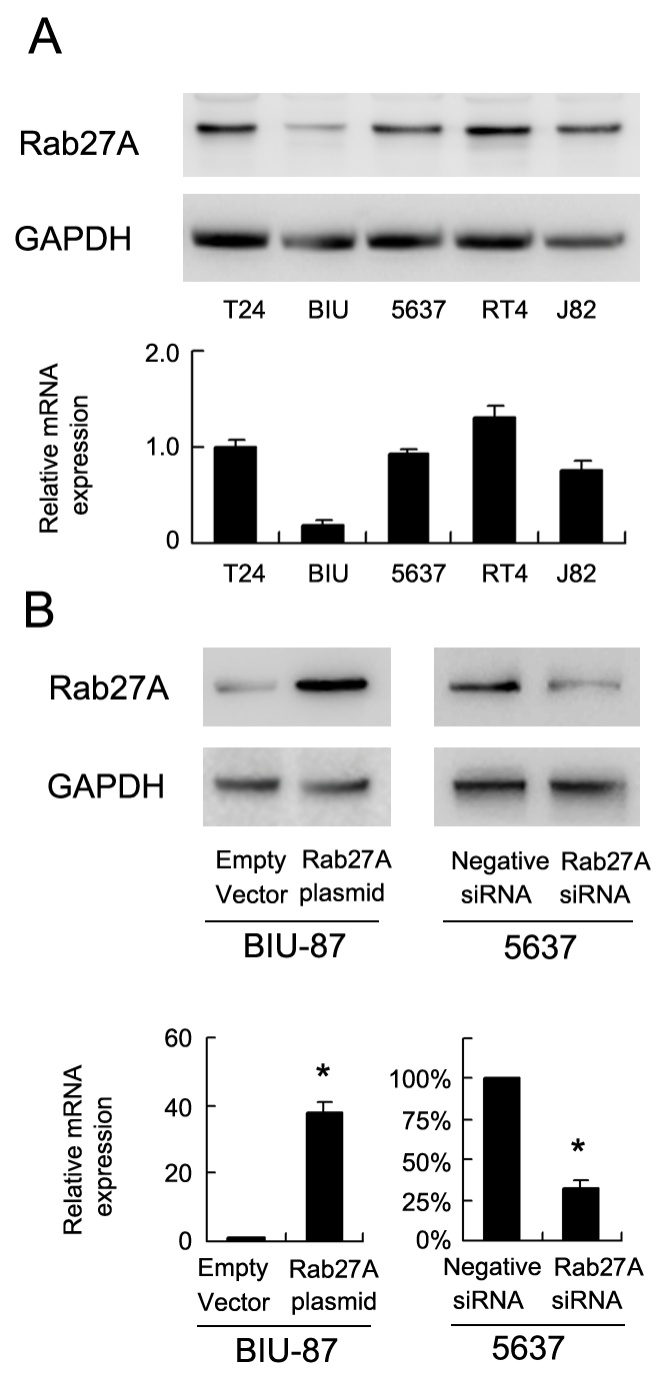
Expression of Rab27A in bladder cancer cell lines **(A)** Western blot and PCR analysis of Rab27A expression in 5 bladder cancer cell lines (BIU-87, 5637, RT4, J82 and T24). **(B)** Western blot and PCR analysis demonstrated that Rab27A siRNA markedly decreases mRNA and protein levels in 5637 cells. Transfection of Rab27A plasmid upregulated its mRNA and protein expression in BIU-87 cell line. * p<0.05.

**Figure 3:**
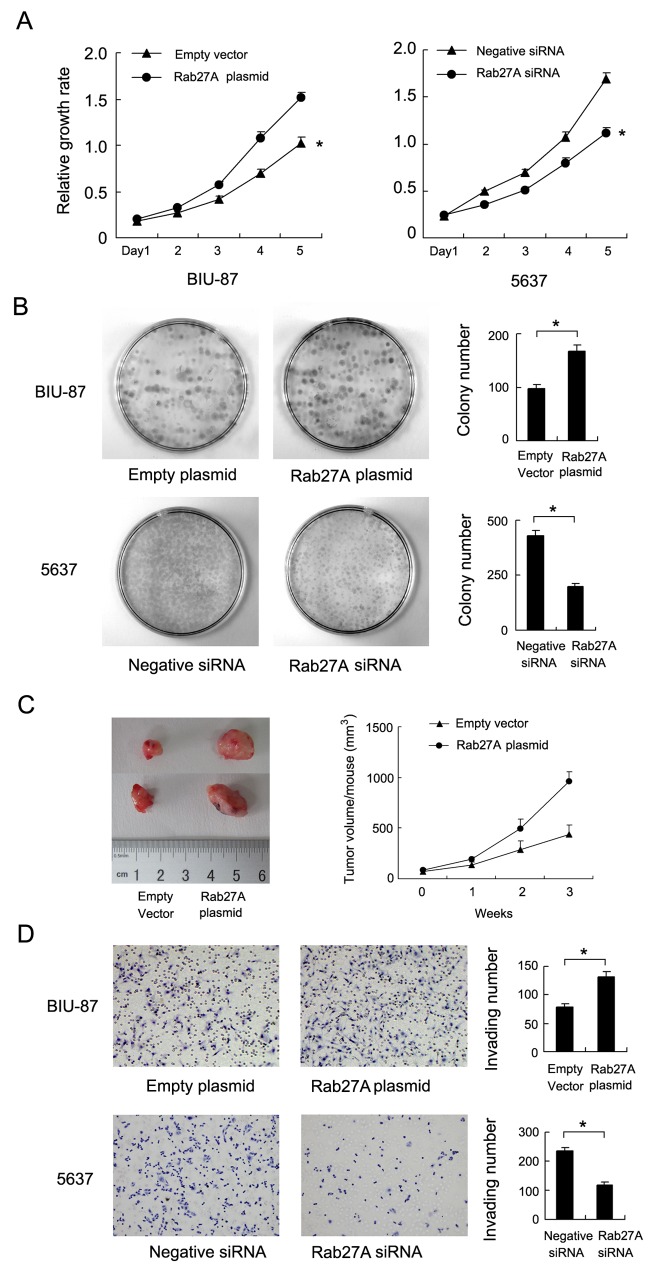
Rab27A promotes proliferation and invasion of bladder cancer cells **(A)** CCK8 assay demonstrated that Rab27A plasmid facilitated cell growth rate in BIU-87 cells while Rab27A siRNA downregulated 5637 growth rate. **(B)** Rab27A plasmid transfection increased colony number of BIU-87 cells. Rab27A siRNA decreased colony number of 5637 cells. **(C)** Nude mice xenografts were sacrificed after 4 week. Tumor volume of BIU-87 transfected with Rab27A was significantly higher than control. **(D)** Matrigel invasion assay showed that Rab27A upregulated invading ability if BIU-87 cells. Rab27A siRNA downregulated invading ability of 5637 cells. * p<0.05.

We checked Rab27A protein in 5 bladder cancer cell lines (5637, RT4, BIU-87, J82 and T24) by western blot and RT-PCR. Relative high Rab27A protein and mRNA was found in 5637, RT4, J82 and T24 while low Rab27A expression was observed in BIU-87 cell line (Figure 2A). To investigate its biological function, we employed Rab27A siRNA in 5637 cell line and Rab27A plasmid in BIU-87 cell line. BIU-87 is a non-muscle invasive bladder cancer cell line. 5637 is a muscle invasive bladder cancer cell line. We confirmed the knockdown and transfection efficiency using western blot analysis and RT-PCR (Figure 2B). CCK-8 assay showed that Rab27A transfection facilirated cell proliferation rate while its depletion inhibited cell growth (Figure 3A). Colony formation assay showed that Rab27A overexpression significant increased colony formation ability of BIU-87 cells (Figure 3B). Rab27A depletion in 5637 cells significantly downregulated colony formation ability. To investigate the effects of Rab27A *in vivo*, we established stable BIU-87 cell line through G418 selection, which was delivered by subcutaneous injection into nude mice. As shown in Figure 3C, Rab27A-transfected BIU-87 cells showed increased growth speed compared with those transfected of empty vector.

Immunohistochemistry results correlated of Rab27 with invading depth, suggesting its potential role in cancer cell invasion. Thus we performed transwell invasion assay to examine the role of Rab27A on the invading ability. The results showed that in Rab27A depleted 5637 cells, the number of invaded cancer cells was significantly decreased. Rab27A overexpression significantly upregulated invading cell number (Figure 3D).

### Rab27A confers cisplatin resistance

**Figure 4:**
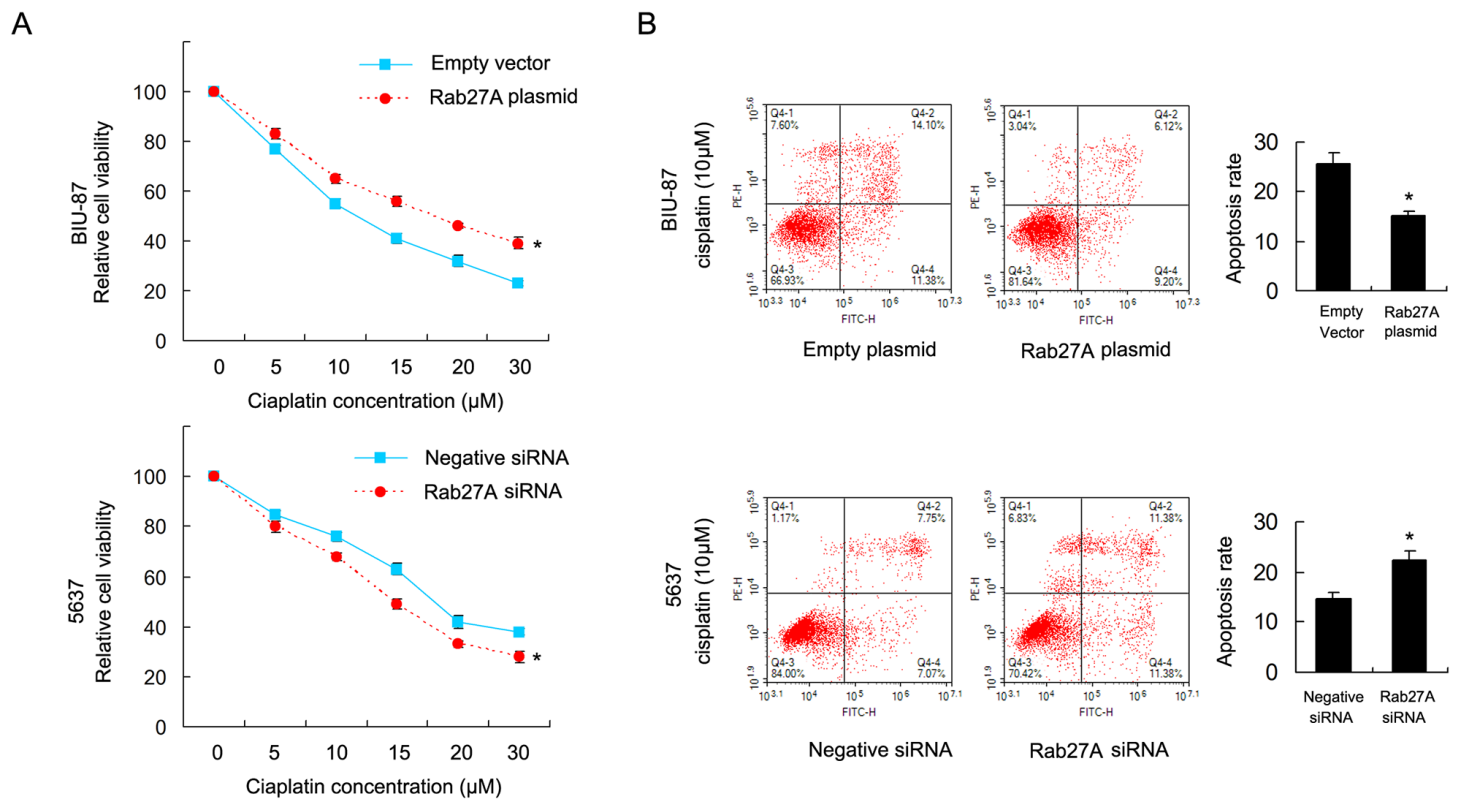
Rab27A confers cisplatin resistance in bladder cancer cells **(A)** CCK8 showed that RAB27A overexpression upregulated cell viability in BIU-87 cells with cisplatin treatment at different concentration. RAB27A siRNA showed the opposite effect by downregulating 5637 cell viability **(B)** Annexin V/PI staining showed that Rab27A plasmid significant downregulated apoptosis rate in BIU-87 cells treated with 10μM cisplatin, while Rab27A siRNA facilitated cisplatin induced apoptosis in 5637 cells. * p<0.05.

To investigate the role of Rab27A on chemoresistance of bladder cancer cells, we used cisplatin to treat cancer cells and adopted CCK8 viability assay to examine survival rate (Figure 4A). Rab27A overexpression maintain cell viability in BIU-87 cells after treatment with cisplatin at different concentration. While Rab27A depletion reduced cisplatin resistance with downregulation of 5637 cell viability.

Annexin V/PI staining was carried out to check apoptosis rate. As shown in Figure 4B, Rab27A overexpression significant downregulated apoptosis rate after 24 hours of cisplatin (10μM) treatment. While Rab27A siRNA upregulated apoptosis rate in 5637 cells treated with cisplatin (10μM). The above results demonstrated that Rab27A could induce cisplatin resistance and inhibit apoptosis in bladder cancer cells.

### Rab27A regulates mitochondrial membrane potential and apoptosis related proteins

**Figure 5:**
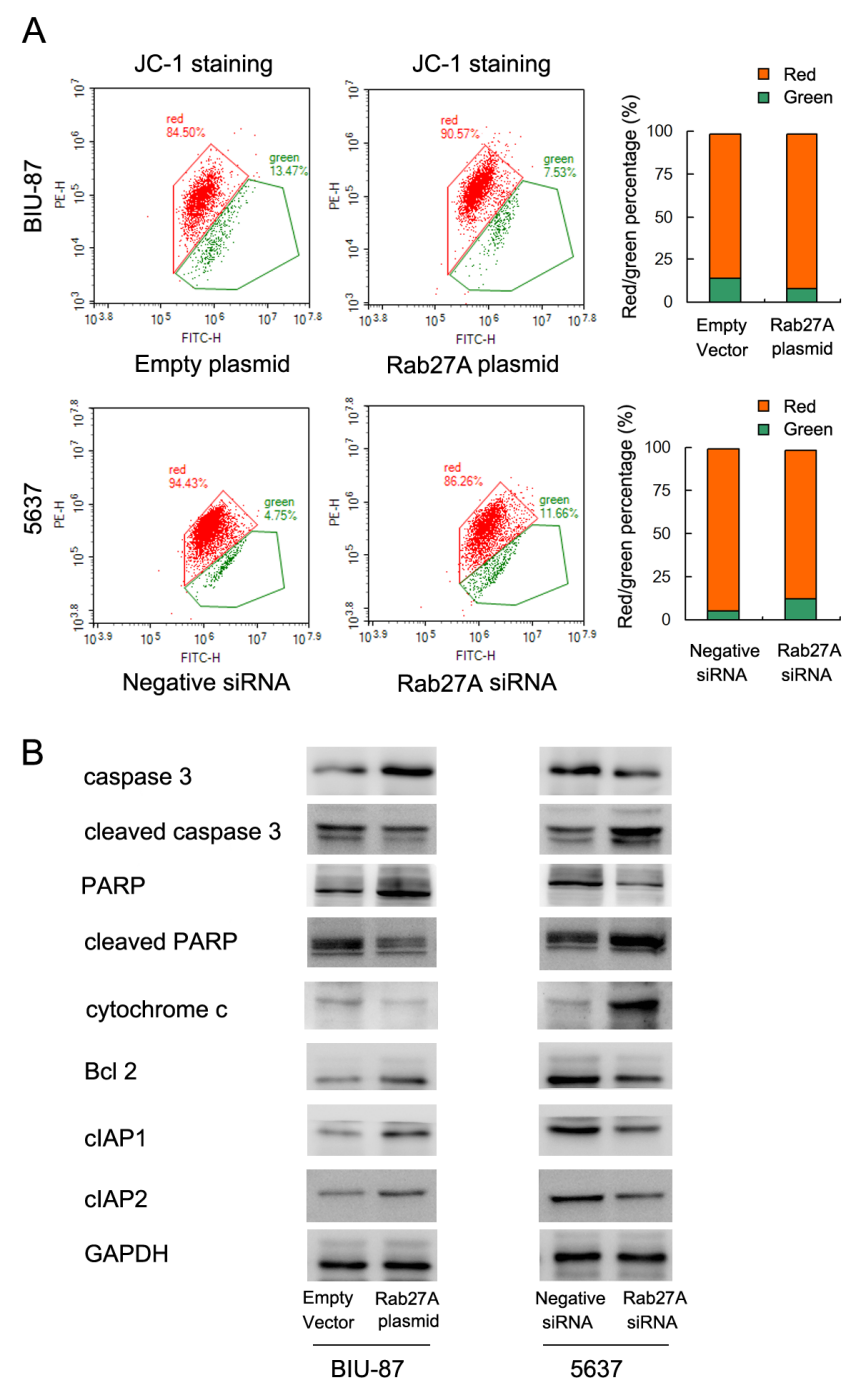
Rab27A regulates mitochondrial membrane potential and apoptosis related proteins **(A)** JC-1 staining and flow cytometry demonstrated that after Rab27A plasmid treatment, the JC-1 red/green ratio increased, indicating upregulated mitochondrial membrane potential. **(B)** Western blot showed that Rab27A overexpression downregulated expression of cytochrome c, cleaved caspase 3 and cleaved PARP while upregulated Bcl-2, cIAP1 and cIAP2 protein. Rab27A siRNA upregulated cytochrome c, cleaved caspase 3 and cleaved PARP while downregulated Bcl-2, cIAP1 and cIAP2 protein.

Mitochondrial function plays an important role during chemotherapy induced apoptosis [14]. Thus we assessed if Rab27A was able to regulate mitochondrial membrane potential (Δψm). Quantitative analysis was carried out using JC-1 staining and flow cytometry. JC-1 would enter mitochondria and form complexes with red fluorescence in cells with high Δψm. While in the circumstance of low Δψm, JC-1 exhibits green fluorescence. When mitochondrial function is impaired, cells exhibit low Δψm and green fluorescence by JC-1 staining [15, 16]. As shown in Figure 5, the percentage of red staining increased when transfected with Rab27A plasmid, suggesting upregulation of Δψm by Rab27A. While in 5637 cells, siRNA downregulated the percentage of red staining cells. These results indicate that Rab27A positively regulate mitochondrial membrane potential in bladder cancer cells.

In addition, we checked several protein related to apoptosis control. We found that Rab27A overexpression downregulated expression of cytochrome c, cleaved caspase 3 and cleaved PARP. Rab27A also upregulated Bcl-2, cIAP1 and cIAP2 protein. While in 5637 cells, Rab27A upregulated cleaved caspase 3, cleaved PARP, cytochrome c and inhibited Bcl-2, cIAP1 and cIAP2 protein (Figure 5B).

### Rab27A regulates cisplatin resistance through NF-κB signaling

**Figure 6:**
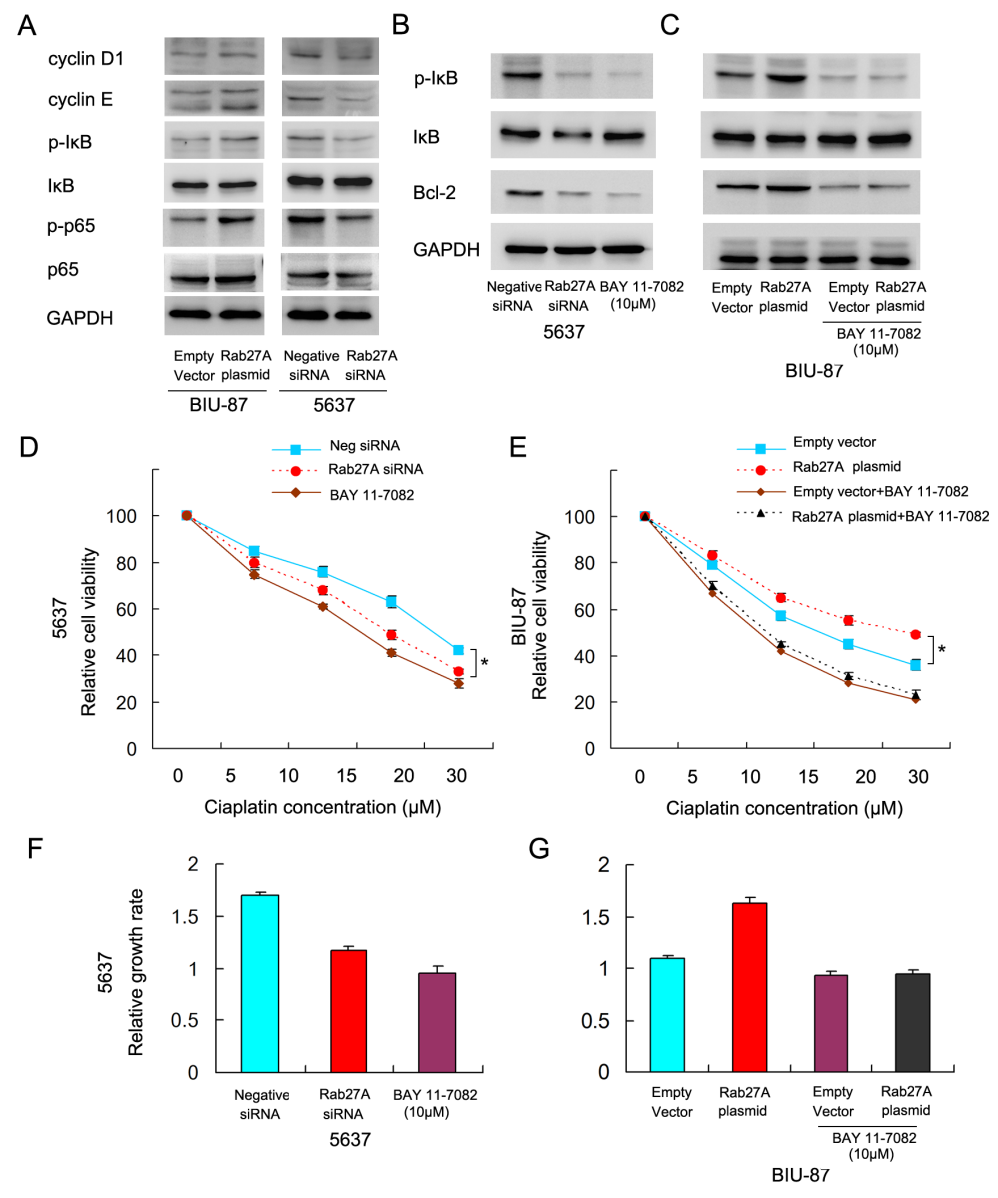
Rab27A regulates cisplatin resistance through NF-κB signaling. **(A)** Western blot showed that Rab27A positively regulated the expression of cyclin E, cyclin D1, p-κB and p-p65. Rab27A siRNA negatively regulated expression of cyclin E, cyclin D1, p-κB and p-p65. **(B)** Bay 11-7082 treatment significantly downregulated IκB phosphorylation and Bcl-2 in 5637 cells. **(C)** Bay 11-7082 significantly downregulated IκB phosphorylation and Bcl-2 in BIU-87 cells. In cells with Bay 11-7082 treatment, Rab27A failed to upregulate Bcl-2. **(D)** Bay 11-7082 treatment downregulated cell viability in 5637 cells. **(E)** Bay 11-7082 treatment abolished the role of Rab27A on upregulation of cell viability in BIU-87 cells. **(F)** NF-κB inhibitor treatment reduced cell growth rate, which was similar to the effect of Rab27A depletion. **(G)** In BIU-87 cells treated with NF-κB inhibitor, Rab27A overexpression failed to upregulate cell growth. * p<0.05.

To investigate the possible mechanism of Rab27A on proliferation, we tested the effect of Rab27A on cell cycle related factors (Figure 6A). Western blot revealed that Rab27A positively regulated the expression of cyclin E, cyclin D1, suggesting Rab27A induces bladder cancer proliferation through cell cycle proteins. NF-κB signaling has been implicated in the regulation of Bcl-2, cIAP1 and cIAP2. We wonder if Rab27A induces chemoresistance through NF-κB pathway. Western blot showed that Rab27A overexpression upregulated p-κB and p-p65 levels while its depletion downregulated p-κB and p-p65 level.

To validate the involvement of NF-κB in Rab27A-regulated cisplatin resistance and Bcl-2 protein, we employed Bay 11-7082 (10μM) which could specifically block IκB phosphorylation. As shown in Figure 6B, 6C, Bay 11-7082 treatment significantly downregulated IκB phosphorylation. In 5637 cell line, Bay 11-7082 treatment downregulated Bcl-2 expression, which was similar to the effect of Rab27A siRNA (Figure 6B). In BIU-87 cell line, Bay 11-7082 abolished the effect of Rab27A on Bcl-2 upregulation (Figure 6C). Furthermore, cell viability assay showed that when treated with cisplatin, blockage of NF-κB downregulated cell viability and abolished the role of Rab27A on upregulation of chemoresistance (Figure 6D, 6E). We also checked the effect of NF-κB on cell proliferation. As shown in Figure 6F, NF-κB inhibitor treatment reduced cell growth rate, which was similar to the effect of Rab27A depletion. In BIU-87 cells treated with NF-κB inhibitor, Rab27A overexpression failed to upregulate cell growth (Figure 6G).

## DISCUSSION

Rab27 protein has been reported in several human cancers. Rab27B promotes invasive growth and metastasis of estrogen receptor (ER)-positive breast cancer cells [7]. Rab27A was identified as a driver gene that provides growth advantage during melanoma progression [8]. Rab27A inhibition in melanoma cell lines reduced primary tumor growth and development of lung metastasis [9]. Rab27A mediated by NF-κB promotes the stemness of colon cancer cells via up-regulation of cytokine secretion [10]. Rab27a expression was significantly associated with grade progression and worse prognosis in all grades of gliomas [11]. These studies suggested Rab27A as a potential onco-protein in human cancers. However, several reports indicates Rab27A as a tumor suppressor. High Rab27A expression indicates favorable prognosis in colorectal cancer [12]. RAB27A and RAB27B are also downregulated in advanced prostate cancer [13]. Thus the expression pattern and role of Rab27A is tissue specific. In this study, we demonstrated Rab27A protein was overexpressed in 44.8% bladder cancer samples. The rate of Rab27A overexpression was higher in MIBC than that in NMIBC, suggesting Rab27A might contribute to tumor invasion and malignant progression of bladder cancer cells.

Next we employed Rab27A specific siRNAs and plasmid to modulate its endogenous expression. CCK-8, colony assay and nude mice xenograft assay demonstrated that Rab27A could promote bladder cancer proliferation *in vitro* and *in vivo*. Cell cycle proteins are most important regulators of cancer proliferation. In addition, both cyclin D1 and cyclin E are NF-κB inducible genes [17, 18]. Thus we checked change of cyclin D1/E and found Rab27A was able to induced cyclin D1 and cyclin E. cyclin D1 and cyclin E play pivotal roles during G1-S checkpoint and their overexpression correlate with malignant growth and aggressive phenotype of bladder cancer cells [19-21]. Matrigel invasion assay also showed that Rab27A positively regulated invading ability. Thus Rab27A promotes both proliferation and invasion of bladder cancer cells, which correlated well with our immunohistochemical data.

Next we checked the role of Rab27A on chemoresistance and cisplatin induced apoptosis. Our data showed that Rab27A upregulated cisplatin resistance with inhibition of apoptosis. Mitochondrial membrane potential is closely associated with development of chemoresistance and apoptosis control. Thus we examined mitochondrial membrane potential (Δψm) using JC-1 staining. We found that Rab27A maintain Δψm while its siRNA decreased Δψm. Loss of Δψm triggers mitochondrial apoptosis through elevated mitochondrial membrane permeability. Thus our data identified Rab27A as a positive regulator of mitochondrial function, which confers resistance to mitochondrial apoptosis in bladder cancer cells.

To further explore the underlying mechanism, we examined related protein and found that Rab27A positively regulates Bcl-2, cIAP1, cIAP2 and inhibits caspase and PARP cleavage. cIAP1/2 belong to the inhibitor of apoptosis protein (IAP) family which consists of an evolutionarily conserved group of apoptosis inhibitors. cIAP1/2 proteins directly interact and inhibit the activity of caspases [22]. Bcl-2 exerts a survival function in response to a wide range of apoptotic stimuli through inhibition of mitochondrial cytochrome c release. It has been implicated in modulating mitochondrial homeostasis. Thus, Bcl-2 may explain elevated mitochondrial membrane potential induced by Rab27A.

NF-κB activation has been reported to play an important part in the development of chemoresistance in various cancers including bladder cancer [23]. The mechanism of NF-κB induced chemoresistance includes induction of XIAP, Bcl-2 and other apoptosis related factors [24]. In addition, NF-κB activation maintains mitochondrial homeostasis and membrane potential, possibly through Bcl family proteins [25]. Our results showed that Rab27A could activate NF-κB signaling by upregulating p-IκB and p-p65 expression. Activation of NF-κB signaling has been report to induce Bcl-2 and IAP family proteins [26, 27]. To confirm the role of NF-κB on Rab27A induced chemoresistance and Bcl-2. We adopted BAY 11-7082, a NF-κB inhibitor, to block NF-κB signaling in bladder cancer cells. The results showed that NF-κB blockage downregulated Bcl-2 protein and abolished the role of Rab27A on chemoresistance, suggesting Rab27A regulates Bcl-2 and chemoresistance through, at least partly, NF-κB activation. Since p65 was sufficient to induce up-regulation of Rab27A and a functional NF-κB binding site in the Rab27A promoter was found, it is possible there is a positive feedback loop between NF-κB and Rab27A. The exact mechanism of Rab27A on NF-κB activation is unclear. Translocation event is essential for gene regulation by NF-κB. Since Rab27A is involved in vesicle transportation, we postulated that Rab27A play a part during NF-κB transportation, which needs further investigation.

In conclusion, our study demonstrated that Rab27A is overexpressed in bladder cancer tissues and promotes cancer proliferation, invasion and chemoresistance, possibly through NF-κB signaling pathway.

## MATERIALS AND METHODS

### Patients and specimens

The study protocol was approved by the institutional reviewer board of Fourth Affiliated Hospital of China Medical University. 87 cases of embedded samples from bladder cancer and corresponding normal adjacent tissues were obtained from pathology archive between 2011 and 2013. Clinical data was obtained from medical records. Inclusion criteria was: Negative restaging Transurethral resection of bladder tumor (TURBT) within 5 months after the first TURBT. Exclusion criteria were:1. presence of concomitant carcinoma in situ; 2. shift from no adjuvant treatment to adjuvant BCG treatment. 12 fresh samples from bladder cancer and corresponding normal adjacent tissues were obtained from patients after surgical treatment. Written informed consent was obtained from all patients and all clinical investigation has been conducted.

### Immunohistochemistry

Immunostaining was performed using the Elivision staining kit from Maixin (Fuzhou, China). Briefly, we used hydrogen peroxide to block endogenous peroxide. Followed by 30 minutes of incubation with normal goat serum, sections were incubated with Rab27A rabbit polyclonal antibody (1:300 dilution; Proteintech, USA) at room temperature for two hours. Then sections were incubated with goat anti-rabbit HRP polymer. DAB plus kit was used for development of staining. We took colorectal cancer tissues as positive control for Rab27A immunostaining. Rabbit IgG isotype antibody was used for negative control. Tumor samples incubated with Rabbit IgG exhibited negative staining. Two investigators examined all tumor sections. 5 views were examined per slide, and 100 cells per view was counted. Cytoplasmic staining was considered as positive. Staining intensity was categorized as follows: 0, negative; 1, moderate; 2, strong. Staining percentage was scored as 0: 0%; 1: 1-25%; 2: 26-50%; 3: 51-75%; 4: 76-100%. Each scores were multiplied to get a final score of 0 to 8. Tumor samples with a final score <4 were regarded as low expression. Tumor samples with a final score of 4-8 were determined as Rab27A high expression. So tumor samples with strong intensity and >25% staining percentage or tumor samples with moderate intensity and >75% staining percentage could be considered as high Rab27A expression.

### Cell culture and transfection

Bladder cancer cell lines BIU-87, J82, T24, 5637 and RT4 were obtained from American Type Culture Collection (Manassas, USA). Cells were cultured in DMEM (Gibco, USA) containing 10% fetal bovine serum (Invitrogen, Carlsbad, CA, USA) at 37°C in 5% CO_2_.

SMARTpool siRNAs for Rab27A and Non-targeting siRNA were purchased from Dharmacon (GE Healthcare, USA). The cells were transfected with siRNAs using the DharmaFECT 1 transfection reagent (GE Healthcare, USA). siRNA purchased from Dharmacon in 5nmol package and diluted in 200ul buffer. For each transfection 10ul diluted siRNA buffer was used.

pCMV6-Rab27A plasmid was obtained from Origene (Origene, Rockville, USA). Attractene Transfection reagent was used (Qiagen, Hilden, Germany). 1.2μg plasmid was used for transfection each time.

### Western blot analysis

Total proteins from cells were extracted in cell-lysis buffer (Pierce, Rockford, IL) and quantified using the Bradford method. About 50 μg protein was transferred to PVDF membranes after separated by SDS-PAGE. The membranes were incubated at 4°C overnight with Rab27A (1:800, Proteintech, USA), cyclin E (4129), cyclin D1 (2978), p-IκB (2859), IκB (4814), p-p65 (3033), p65 (8242), caspase 3 (9668), cleaved caspase 3 (9664), PARP (9532), cleaved PARP (5625), cytochrome c (11940), Bcl-2 (15071) (1:1000; Cell signaling, Boston, USA) and GAPDH (1:2000; Santa Cruz, USA). After incubation with HRP-coupled anti-mouse/rabbit IgG antibody (1:2000 dilution, Cell Signaling Technology, USA) at 37°C for 2 hours. Target proteins on PVDF membrane were visualized using ECL (Pierce, USA) and captured using a DNR BioImaging System (DNR, Israel).

### Realtime PCR

RNA extraction was performed using RNAiso (TAKARA, China).

Realtime PCR was performed using SYBR Green MasterMix from ABI (Applied Biosystem, USA). Realtime PCR was carried out using ABI 7500 (Applied Biosystems, USA). A dissociation procedure was performed to generate a melting curve for confirmation of amplification specificity. β-actin was used as the reference gene. Relative quantification of target genes was calculated using the 2^-ΔΔCt^ method.

### CCK-8 assays

Cell proliferation/cell viability speed was analyzed using Cell Counting Kit-8 (CCK-8) kit (Dojindo, Gaithersburg, MD) according to the manufacturer’s protocol. 48 hours after transient plasmid transfection, cells were seeded about 5×10^3^ in 96-well plates. Every day the cells were treated with 10 μl CCK-8 solution for 4 hours in the CO2 incubator. The 96-well plates were measured at 450 nm.

### Colony formation assay

After transfection for 48 hours, cells were seeded into three 6 cm cell culture dish and incubated for about at least two weeks. Then the plates were washed with PBS and stained with Giemsa. Colony number was counted manually.

### Annexin V/PI analysis

For determination of apoptosis, Annexin V/PI kit (BD bioscience) was used to stain cells. Cells were harvested by 0.25% trypsin, washed twice with chill PBS, followed by being resuspended in 250 μl of binding buffer. Staining solution containing Annexin V/FITC and propidium iodide was added in cell suspension. The stained cells were measured by ACEA Flow Cytometer (ACEA, USA). Data was analyzed using Novoexpress software (ACEA, USA).

### Mitochondrial membrane potential

The mitochondrial membrane potential (Δψm) was detected by using JC-1 staining method. Briefly, cells were harvested, washed with PBS and incubated with 5 μM JC-1 (Cell Signaling Technology) for 30 minutes in the incubator. Then cells were washed and analyzed using a ACEA flow cytometer (ACEA, USA). Data was analyzed using Novoexpress software (ACEA, USA).

### Mouse xenograft study

The use of animal was approved by the Animal Care and Ethics Committee of China Medical University and was in line with the Guide for Care and Use of Laboratory Animals of China Medical University. Female athymic nude mice were purchased were purchased from Shanghai Slac Laboratory Animals Ltd. Selection of stable BIU-87/Rab27 and BIU-87/Empty vector cell lines were accomplished with G418 (Sigma) at a concentration of 0.2mg/mL. A xenograft model was established by subcutaneous right armpit injections of stable cell lines (1*10^7^). Tumor size was measured every week. After 4-5 weeks growth, mice were sacrificed and tumors were removed.

### Statistical analysis

SPSS 17 for Windows was used for all statistical analyses. χ2 test was used to examine possible correlations between Rab27A expression and clinicopathologic factors. p<0.05 was considered as statistical significance.
